# Track Deflection Monitoring for Railway Construction Based on Dynamic Brillouin Optical Time-Domain Reflectometry

**DOI:** 10.3390/s24248205

**Published:** 2024-12-23

**Authors:** Tianfang Zhang, Liming Zhou, Weimin Liu, Linghao Cheng

**Affiliations:** Guangdong Provincial Key Laboratory of Optical Fiber Sensing and Communications, Institute of Photonics Technology, Jinan University, Guangzhou 510630, China; tfzhang1008@163.com (T.Z.); limingok@126.com (L.Z.); ll520lwm@163.com (W.L.)

**Keywords:** distributed fiber optic sensing, Brillouin optical time domain reflectometry, track deformation monitoring

## Abstract

Real-time online monitoring of track deformation during railway construction is crucial for ensuring the safe operation of trains. However, existing monitoring technologies struggle to effectively monitor both static and dynamic events, often resulting in high false alarm rates. This paper presents a monitoring technology for track deformation during railway construction based on dynamic Brillouin optical time-domain reflectometry (Dy-BOTDR), which effectively meets requirements in the monitoring of both static and dynamic events of track deformation. Dy-BOTDR can provide a two-dimensional spatial–temporal distribution map of track strain changes to characterize various events for better monitoring accuracy and lower false alarm rates.

## 1. Introduction

With the rapid development of the economy, dense railway networks inevitably face a large amount of renovation and expansion. To avoid significant disruptions to socio-economic operations, these railway constructions often require railway lines to continue to operate without interruption so that trains can pass efficiently and safely during construction [1]. Therefore, during railway construction, the track’s smoothness must be maintained below the safety control values for train operation [2]. This necessitates the real-time online monitoring of track deformation during construction for the timely detection of any excessive deformation to prevent disaster events [3].

Total stations are widely used high-precision surveying instruments for above-ground large constructions and underground tunnel projects [4]. They can monitor track displacement to determine track deformation [5]. However, the low data refresh rate of total stations is too low for real-time monitoring and timely alarms [6]. Additionally, total stations are significantly affected by weather conditions, making all-weather operation challenging [7]. Given these limitations, distributed optical fiber sensing (DOFS) technology, as an emerging and highly promising monitoring method, has gained widespread attention in recent years [8]. DOFS can operate stably under extreme conditions and is known for its high sensitivity and excellent real-time performance [9]. It allows for the continuous, real-time monitoring of track deformation and can quickly issue alarms when abnormal deformation is detected [10], providing more reliable and efficient technical support for railway construction and maintenance [11].

Phase-sensitive optical time domain reflectometry (phi-OTDR) is a technology widely studied and applied in railway monitoring [12]. It is highly sensitive and responsive to vibrations and acoustic waves, enabling the monitoring of the railway and its surroundings through vibrations and sounds, thereby analyzing faults, disasters, and other events [13]. However, this technology is generally suitable for monitoring vibrations and sounds at frequencies above tens of hertz, making it less suitable for static deformation monitoring [14]. Furthermore, its spatial resolution is typically at a scale of ten meters [15], which may not meet the requirements for track deformation monitoring. Moreover, power fading in phi-OTDR is also a problem [16]. In fact, phi-OTDR is more suitable for monitoring dynamic events with certain real-time requirements and is not well suited for static events. Optical frequency-domain reflectometry (OFDR) is another promising technique that provides a spatial resolution down to the sub-millimeter. But its measurement speed is still slower than that of the Dy-BOTDR reported in this paper. Moreover, the measurement range of OFDR is limited by the coherence length of the laser source and is normally quite short [17]. Fiber Bragg grating sensors can monitor static deformations and achieve spatial resolutions below one meter [18]. However, the installation and maintenance of these sensors on railway tracks are quite difficult [19].

This paper introduces a track deformation monitoring technology during construction based on dynamic Brillouin optical time-domain reflectometry (Dy-BOTDR). The uniqueness of BOTDR lies in its transceiver architecture, which only requires signal transmission and reception at one end of the sensing fiber, thereby demonstrating exceptional resilience against events such as fiber breakage in practical engineering applications [20]. Among the technical solutions utilizing the Brillouin scattering phenomenon to implement reflectometers, most of the reported methods capable of monitoring dynamic events are based on BOTDA [21], while reports on Dy-BOTDR are still rare. However, implementations of BOTDA require a closed fiber loop, which is unfavorable in practical engineering due to fiber faults such as breaks. If BOTDR could achieve dynamic monitoring, its single-ended operation would greatly facilitate engineering deployment and represent a significant improvement for practical engineering applications. Dy-BOTDR technology achieves a significant increase in measurement speed by introducing innovative signal processing methods, with data refresh rates reaching the hertz level, up to as high as 100 Hz. This breakthrough significantly expands the monitoring capability of the technology for signals ranging from static to dynamic signals up to tens of hertz. This feature provides strong support for track deformation monitoring in construction environments, allowing the system to more effectively differentiate and identify various types of track deformation events that occur during construction [22], thereby significantly improving analysis accuracy and greatly reducing the likelihood of false alarms.

In optical fibers, the power and frequency shift in Brillouin scattering light have a linear relationship with changes in temperature and stress [23]. Based on this physical principle, BOTDR technology enables the distributed sensing of temperature and strain along the optical fiber.

The core of BOTDR technology lies in the precise analysis of the Brillouin scattering spectrum, especially the extraction of its central frequency—the Brillouin frequency [24]. However, traditional methods of scattering spectrum analysis, such as using narrow-band filters to scan each frequency point and obtain the entire power spectrum of the scattered light, are limited in real-time performance due to their large computational load and significant time consumption [25]. To overcome this bottleneck, strategies based on wideband signal processing have been introduced, particularly utilizing Short-Time Fourier Transform (STFT) combined with Lorentzian fitting, which can quickly reconstruct the complete power spectrum of the scattering light [26]. Although this method effectively improves processing speed, the high measurement precision that accompanies it results in high computational complexity, which still limits further efficiency gains. The higher the measurement precision of the Brillouin frequency achieved by STFT, the higher the computational complexity, leading to increased resource consumption and computation time, thus becoming a major obstacle to improving measurement speed [27].

In light of this, we propose an innovative method based on instantaneous frequency analysis, which cleverly uses the instantaneous phase changes between adjacent sampling points in the time domain to directly estimate the Brillouin frequency [20]. This approach significantly reduces the computational complexity of Brillouin frequency analysis and greatly shortens both the computational complexity and overall measurement time. Within this technical framework, we achieve a BOTDR system with a spatial resolution of 1.2 m over a measurement distance of 5 km. Experimental data indicate that this new method achieves a standard deviation of 0.5065 MHz for the measurement of Brillouin frequency during a single measurement that includes 4096 averages. This precision is comparable to that of the traditional algorithms based on STFT and Lorentzian fitting. Crucially, however, the new method achieves a 122-fold reduction in computational complexity, thereby reducing the single measurement time to 2.06 s, whereas the traditional algorithm takes 251.18 s. This demonstrates the significant potential of this method to enhance measurement efficiency.

The BOTDR based on instantaneous frequency analysis lays the foundation for achieving Dy-BOTDR, ultimately enabling BOTDR to measure not only static strain and temperature but also physical quantities such as dynamic strain that change at low frequency. This capability provides the possibility of distinguishing between dynamic and static events in practical engineering applications, thereby improving the accuracy of event analysis.

## 2. Methods

BOTDR can obtain strain data of the rail at the fiber surface by detecting the Brillouin frequency shift caused by axial strain in the fiber. However, this strain data cannot directly reflect the bending of the rail due to vertical loads; further analysis and processing are needed to provide a more intuitive representation of the rail’s condition [28]. This processing involves two parts: the derivation of the mathematical conversion from strain to bending and the feature extraction of the strain data [29].

**Figure 1:**
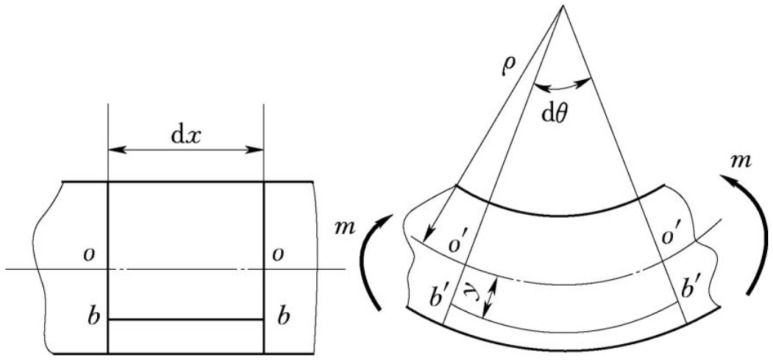
Schematic diagram of the beam micro-element before and after deformation.

A simply supported beam model can be used to study the structural unit of a section of a rail positioned between two sleepers. For a micro-element dx of the beam (as shown in Figure 1), assume that the horizontal direction is the x-axis and the longitudinal direction is the y-axis when the rail is horizontal. The neutral axis before deformation is denoted as oo, and after deformation it is $o^{\prime }o^{\prime }$. The position of the optical fiber is bb, and after deformation, it is $b^{\prime }b^{\prime }$. It is assumed that the deformation of the optical fiber is synchronized with the deformation of the rail surface, meaning that the strain in the optical fiber is equal to the strain in the rail surface. The distance from the optical fiber’s position to the neutral axis is $y_{0}$, and this distance remains unchanged before and after deformation [30].

The infinitesimal arc angle of the cross-sections on both sides of the micro-element after deformation is dθ, and the radius of curvature to the neutral axis is ρ. Therefore, the length of the optical fiber after deformation is given by the following:

(1) $$b^{\prime }b^{\prime }=(\rho +y_{0})d\theta$$

The length of bb is equal to oo. Since dθ is very small, the length of the neutral axis during the bending process can be considered constant; thus,

(2) $$bb=oo=o^{\prime }o^{\prime }=\rho d\theta$$

Therefore, the strain of the optical fiber at this micro-element can be expressed as follows:

(3) $$\varepsilon (x)=\frac{b^{\prime }b^{\prime }-bb}{bb}=\frac{(\rho +y_{0})d\theta -\rho d\theta }{\rho d\theta }=\frac{y_{0}}{\rho }$$

The parameters change as the rail deforms, and the deformation ε(x) is transformed into the curvature radius of each micro-element. We can use the curvature radius ρ to determine the deflection curve of the rail.

The deflection curve describes the function curve of the neutral axis distribution within the beam’s plane, denoted as follows:

(4) y=ω(x)

According to the rules of calculus [31], the curvature of a curve at any given point is calculated using the following formula:

(5) $$\frac{1}{\rho }=\pm \frac{\omega^{\prime \prime }}{{\left[1+{\left(\omega^{\prime }\right)}^{2}\right]}^{\frac{3}{2}}}$$

At this point, we can combine the formula with Equation (3) to establish the relationship between the strain in the rail and the deflection curve:

(6) $$\varepsilon (x)=\pm \frac{\omega^{\prime \prime }}{{\left[1+{\left(\omega^{\prime }\right)}^{2}\right]}^{\frac{3}{2}}}y_{0}$$

The positive and negative signs indicate the position of the curvature radius. Here, we set the strain as positive when it is elongating, which means the beam is deflected downward; conversely, when the strain is compressive, it is negative, indicating the beam is arching upward. Therefore, we will omit the explicit positive and negative signs in the subsequent calculations. Equation (6) represents a second-order nonlinear ordinary differential equation and can be simplified. Since the value of $\omega^{\prime }$ in the denominator is very small compared to 1 [32], (6) can be approximated as follows:

(7) $$\frac{d^{2}\omega }{dx^{2}}=\frac{\varepsilon (x)}{y_{0}}$$

It then shows that the deflection curve can be deduced by integrating the strain data obtained by BOTDR twice. However, the discrete deformation data provided by BOTDR contain a significant amount of noise [33], which is highly sensitive to integration. Direct integration cannot accurately reflect the deflection curve of the track. Therefore, it is necessary to process the deformation data to reduce the impact of noise before the integration process.

A segment of steel rail currently under bridge construction has been selected as the experimental testing target to demonstrate the capability of Dy-BOTDR in the monitoring of static and dynamic events. The tested railway is a single-track, non-electrified line with a maximum operating speed of 120 km/h. It features 60 kg/m steel rails, continuous track technology, reinforced concrete sleepers, and a ballast bed structure, with a curve radius of 400 m and a cant design of 125 mm.

## 3. Results and Discussion

**Figure 2:**
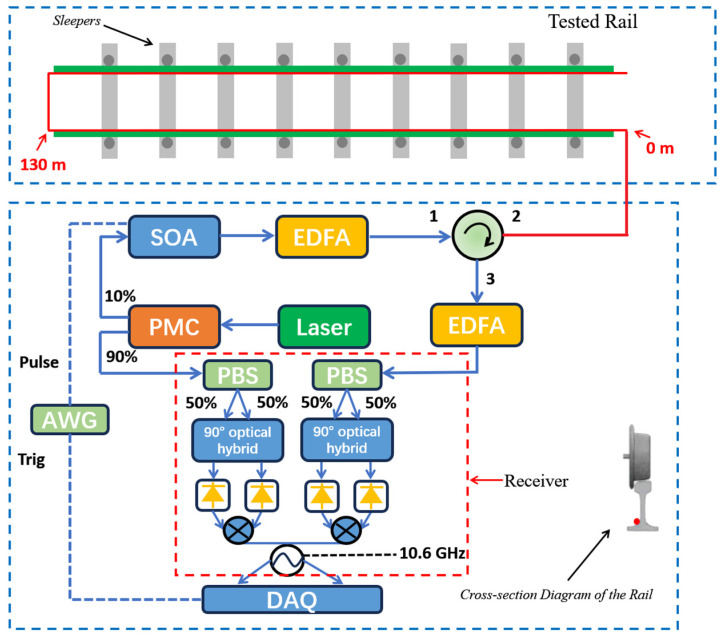
Real-time online monitoring system for track deformation of the tested railway based on dynamic BOTDR. PMC: polarization maintain coupler; SOA: semiconductor optical amplifier; EDFA: erbium-doped fiber amplifier; AWG: arbitrary waveform generator; DAQ: data acquisition.

The track deformation monitoring system, as shown in Figure 2, is equipped with strain sensing optical fibers installed at the interface between the rail and the rail bed along a track. The schematic diagram of the dynamic BOTDR system is shown in the lower part of Figure 2. The laser source is a narrow-linewidth laser with a linewidth less than 100 kHz and an output power of 20 mW. The output of the laser source is split into two parts with one for the receiver as the local oscillator and the other for pulse modulation by a semiconductor optical amplifier (SOA). The receiver is a 100 G integrated coherent receiver (ICR) supplied by Finisar, which consists of an integrated polarization beam splitter and four balanced photoreceivers monolithically integrated with optical 90° hybrids. The SOA from INPHENIX works in switching mode to gate the laser output to pulses which are then amplified by an erbium doped fiber amplifier (EDFA) to a peak pulse power of about 300 mW. The pulse output of EDFA is directed to the sensing fiber on the rail track through an optical circulator, and the Brillouin scattering from the fiber is amplified by another EDFA before entering the ICR. Inside the ICR is a polarization diversity coherent receiver. The Brillouin signal output from the ICR is at around 11 GHz, which is then down-converted by microwave mixers to less than 500 MHz before digitization and acquisition (DAQ) for further digital processing. The specific location of fiber installation is marked as the red dot in the cross-sectional diagram of a rail in Figure 2. The length of the rail track under monitoring is about 130 m. The optical fibers on the dual tracks are connected in a series to form a single-chain monitoring network, ensuring data continuity and integrity. The monitoring system establishes a high-density data collection point every 0.1 m, resulting in over 5000 measurement points along the entire track, providing comprehensive coverage of the monitoring area. The system continuously refreshes the monitoring data at a frequency of 1 Hz, ensuring real-time accuracy and reliability.

**Figure 3:**
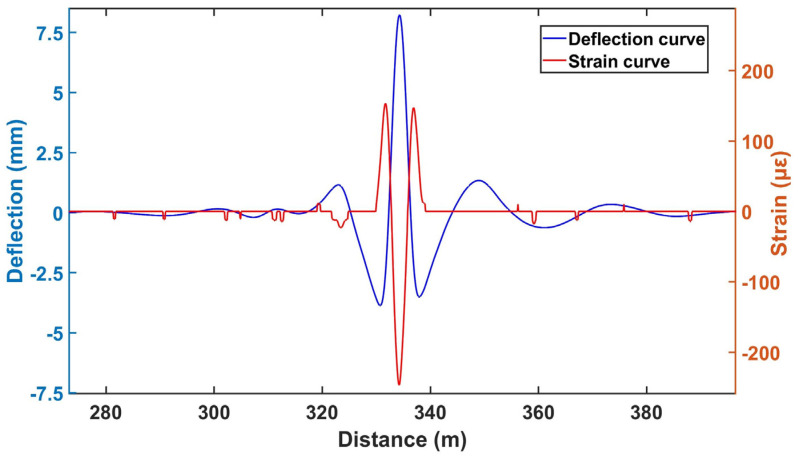
The deflection and strain curve at a specific location on the railway track.

A lifting test at certain a position was conducted to verify whether the monitoring system could effectively, and in real-time, sense track changes and promptly issue alarm signals upon detecting abnormalities. Figure 3 shows a strain and the corresponding deflection curve along the rail obtained in a rail lifting test. The deflection curve is calculated from the strain data directly measured by BOTDR, followed by wavelet analysis for denoise and then integration.

**Figure 4:**
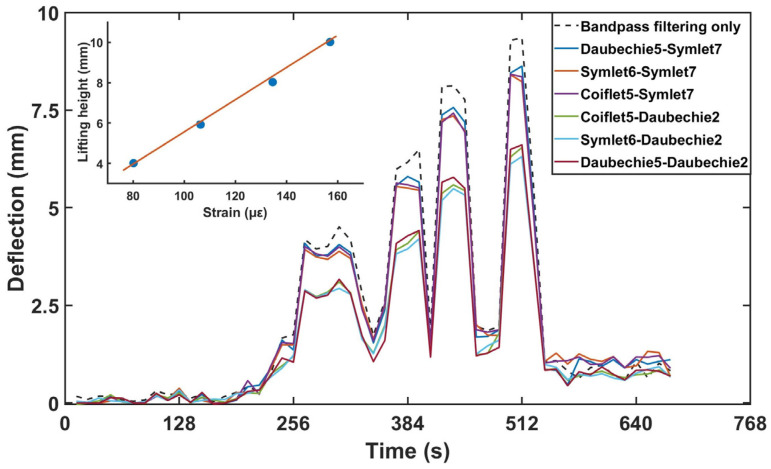
Maximum values of the deflection curve processed with different wavelet functions.

Dynamic BOTDR can directly restore the deformation of the rail, but this is not sufficient to reconstruct the rail’s deflection. In fact, the process of deriving the deflection from the deformation curve is detailed in Equations (3)–(6) in Section 3, where it can be seen that the deflection is the second integral of the deformation curve. The results in Figure 4 are a reconstruction of the deformation data measured along the entire fiber.

**Figure 5:**
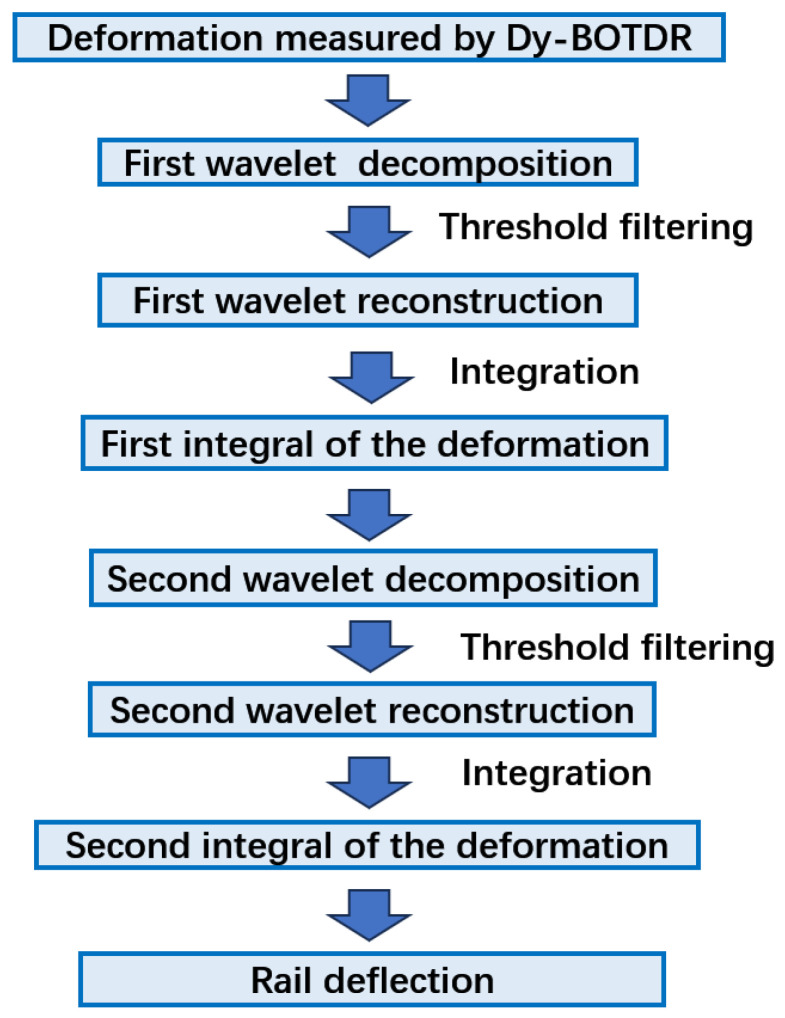
Algorithm flow for calculating rail deflection from deformation.

However, integration is sensitive to noise in data, which leads to the significant drifting of the results. Directly integrating the noisy discrete deformation data is hence not reliable. Therefore, we employ wavelet transform and analysis to denoise the data before reconstruction through integration, and this process is shown in Figure 5. However, this processing method is based on a physical model and produces theoretical results only. In reality, many physical parameters of the rail track are hard to obtain, such as Young’s modulus, the neutral axis and so on. Fortunately, the physical model gives a linear relationship between the strain and deflection of the track. Therefore, we can calibrate the measured strain data to the deflection of the track through a lifting experiment. In the experiment, a track is lifted to a height and the maximum strain measured by BOTDR is then recorded. In this way, a linear relationship between maximum strain and height is established and the slope of this linear relationship is obtained. A theoretical slope calculated through a physical model based on some ideal physical parameters is also obtained and is found to be quite close to the result of the calibration except for a small-scale constant. Later in field monitoring, the slope is used to deduce the maximum track deflection in real time.

A linear mathematical relationship between the deformation curve and deflection after reconstruction is then established. For comparison, we used several different types of wavelet analysis methods, which are all shown in the results in Figure 4. Lifting tests have been carried out several times at different lifting heights. The measured lifting height versus time at the lifting position is shown in Figure 4, indicating 4 lifting events of about 4 mm, 6 mm, 8 mm and 10 mm, respectively. In this figure, different-colored lines indicate results after denoising by different wavelet functions, while the dashed line represents the results obtained from integration being carried out twice after bandpass filtering only, without wavelet analysis. Additionally, comparing these with the inset in Figure 4 reveals that the maximum deflection correlates linearly with the maximum strain. It then shows that the calculated lifting height from the strain data is sensitive to the denoise method. However, the linear relationship between the strain and the height is still retained. The height may be quickly deduced from the strain data through a coefficient obtained by field trials.

Relying solely on the spatial distribution or temporal evolution of track strain can quickly capture changes in deformation and enable preliminary alarm functions. However, this one-dimensional data analysis method has its limitations. Specifically, it fails to fully utilize the unique spatiotemporal advantages of distributed sensing technology, making it difficult to accurately distinguish between different types of strain events in complex environments. For instance, the deformation caused by a train passing normally may exhibit similar characteristics to certain abnormal deformations when analyzed in a single dimension, leading to potential misjudgments and false alarms. In contrast, dynamic BOTDR technology offers rapid strain monitoring capabilities, providing a more reliable technical foundation for extracting and analyzing the spatiotemporal characteristics of strain events.

**Figure 6:**
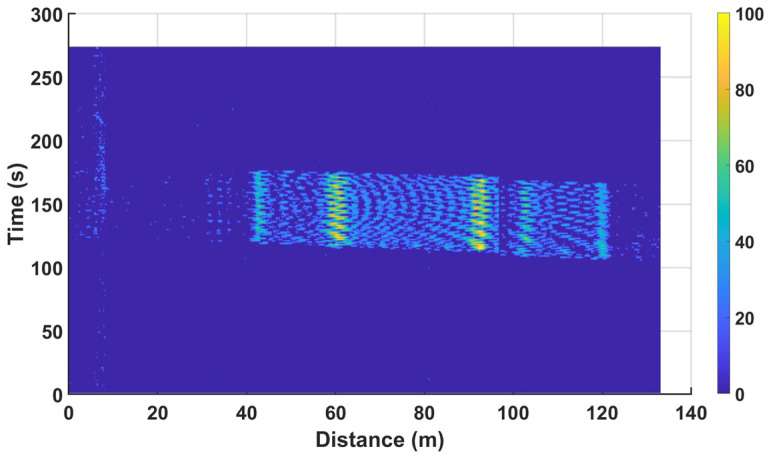
Real-time spatiotemporal two-dimensional distribution map of track strain changes as the train passes.

Figure 6 shows a spatiotemporal two-dimensional distribution map of the track strain change output by the dynamic BOTDR monitoring system during a train passing through a construction section. The map clearly indicates a distinct two-dimensional strain image trajectory in the construction area as the train passes, reflecting the track deformation caused by the train, while the non-construction area maintains a relatively stable strain state, highlighting the contrast between the two. This phenomenon aligns with the actual structural support strength on-site, where the strain changes in the construction area are significantly greater than those in the non-construction area.

Additionally, the spatiotemporal distribution map provides richer information. The motion of the train creates a band-shaped trajectory with a slope in the two-dimensional distribution map due to the changes in track strain. By calculating the slope of this band trajectory, the train’s speed while passing is determined to be 33.73 km/h. Multiplying this speed by the duration of the trajectory allows for an estimation of the train’s length to be approximately 442 m.

It is noteworthy that in the trajectory image, there are two locations where the strain change significantly increases, indicating that the structural support strength in these areas is weaker than others. These should be prioritized for subsequent maintenance and inspection. Additionally, after the train passes through the construction section, the track strain returns to normal values, demonstrating the good resilience and stability of the track structure. In summary, the spatiotemporal two-dimensional distribution map not only provides rich information about the train’s operational status and track deformation characteristics but also offers strong technical support for the timely detection and resolution of potential structural issues.

## 4. Conclusions

This paper introduces an innovative real-time online monitoring technology for track deformation during construction based on Dy-BOTDR. This technology, with its outstanding spatial resolution, rapid response speed, and ability to provide detailed spatiotemporal distribution characteristics of events fully meets the requirements for distinguishing dynamic and static events in track deformation monitoring during construction.

In multiple engineering case studies, this technology has demonstrated the ability to update the spatiotemporal distribution of strain changes in real time at a measurement frequency close to 1 Hz, even over a measurement range of up to 5 km. Whether measuring slight upward or downward deformations of the track, dynamic strain changes during high-speed train passages, or the complexities of framework insertion construction, the technology has proven effective. This capability not only ensures precise measurement of strain changes and track displacement but also expands its application scope, allowing it to provide additional valuable data such as train speed, length during passage, and the distribution of structural support strength within the construction area.

In summary, the application of this technology provides richer and more comprehensive data support for monitoring track deformation during construction, significantly enhancing the timeliness and accuracy of warnings, thereby offering robust technical assurance for ensuring safe and efficient railway construction operations.

## Data Availability

Data are available upon request by contacting the corresponding author.
